# Identifying a novel KLF2/lncRNA SNHG12/miR-494-3p/RAD23B axis in Spare Nerve Injury-induced neuropathic pain

**DOI:** 10.1038/s41420-022-01060-y

**Published:** 2022-05-27

**Authors:** Jinyuan Zhang, Hanping Zhao, Aimin Zhang, Chengyi Zhao, Zhi Mei, Haiyan Yao, Zhidan Fan, Daochen Liang

**Affiliations:** 1 Department of Orthopedics Surgery, Zhongshan People’s Hospital, Zhongshan, China; 2 Department of Anatomy, Southern Medical University, Guangdong Provincial Key Laboratory of Medical Biomechanics, Guangzhou, China

**Keywords:** Cellular neuroscience, Gene regulation

## Abstract

Spinal cord injury (SCI) is a devastating condition for patients, affecting nearly 2.5 million people globally. Multiple side effects of SCI have resulted in a terrible life experience for SCI patients, of which neuropathic pain has attracted the most scientific interest. Even though many efforts have been made to attenuate or eliminate neuropathic pain induced by SCI, the outcomes for patients are still poor. Therefore, identifying novel diagnosis or therapeutic targets of SCI-induced neuropathic pain is urgently needed. Recently, multiple functions of long non-coding RNA (lncRNA) have been elucidated, including those in SCI-induced neuropathic pain. In this study, lncRNA small nucleolar RNA host gene 12 (SNHG12) was found to be upregulated in the dorsal root ganglion (DRGs) of rats with spare nerve injury (SNI). By constructing SCI rat models, we found that lncRNA SNHG12 expression was increased in the DRGs, and mainly distributed in the cytoplasm of PC12 cells. Paw withdrawal threshold (PWT), paw withdrawal latency (PWL), and enzyme linked immunosorbent assay (ELISA) results indicated that lncRNA SNHG12 knockdown attenuated SNI-induced neuropathic pain, and decreased the expression levels of interleukin (IL)−1β, IL-6, and tumour necrosis factor α (TNF-α) in the DRGs. Bioinformatics analysis, RNA pull-down, chromatin immunoprecipitation (ChIP), and luciferase reporter gene assays showed that lncRNA SNHG12 regulates the RAD23 homologue B, nucleotide excision repair protein (RAD23B) expression, through targeting micro RNA (miR)−494-3p. Furthermore, the study indicated that Kruppel-Like Factor 2 (KLF2) could regulate lncRNA SNHG12 expression in PC12 cells. This study identified a novel KLF2/lncRNA SNHG12/miR-494-3p/RAD23B axis in SNI-induced neuropathic pain, which might provide a new insight for developing novel diagnosis, or therapeutic targets of SCI-induced neuropathic pain in the future.

## Introduction

Spinal cord injury (SCI) is a considerable burden on global health care systems, influencing nearly 2.5 million people. SCI is associated with many side effects such as the dysfunction of the body’s motor, sensory, autonomic, and reflex functions [1]. Moreover, the neuropathic pain resulting from SCI, which is characterised by spontaneous pain, allodynia, and hyperalgesia, affects 85% of patients and its incidence is increasing every year [2–4]. Despite the significant efforts made in the past decades to attenuate the health burden from neuropathic pain inflicted on SCI patients, the molecular mechanisms underlying the development of neuropathic pain are still unclear. Therefore, identifying novel diagnosis and therapeutic targets for neuropathic pain is urgently needed.

Long non-coding RNAs (lncRNA), with a length of approximately 200 nucleotides, have been studied in-depth since the technological innovation of high through-putting sequencing [5]. Mechanically, lncRNA is able to alter protein expression in many ways, such as modifying chromatin, influencing transcription, and regulating the decay process of mRNA and subcellular protein distribution [6, 7]. LncRNA can modulate multiple cellular processes, such as cell proliferation, apoptosis, migration, invasion, angiogenesis, inflammation, and immune response [8–12]. In the past decade, multiple functional lncRNAs have been identified in neuropathic pain research. LncRNA MALAT1 is involved in the progression of neuropathic pain by acting as a molecular sponge for micro RNA (miRNA) [13, 14]. LncRNA SNHG1 alleviates neuropathic pain induced by SCI by modulating the expression level of CDK4 [15]. LncRNA NEAT1 contributes to the neuropathic pain induced by SCI via the miR-128-3p/AQP4 axis [16]. Based on this evidence, we concluded that lncRNA is a promising direction for developing novel diagnosis or therapeutic targets for SCI-induced neuropathic pain.

LncRNA small nucleolar RNA host gene 12 (SNHG12) has been studied in-depth in many pathological processes, including glioblastoma, cervical cancer, pancreatic cancer, renal cell carcinoma, and gastric cancer [17–21]. In the nervous system, Long et al. demonstrated that lncRNA SNHG12 could ameliorate brain microvascular endothelial cell injury via sponging of miR-199a. However, whether lncRNA SNHG12 modulates neuropathic pain remains unaddressed.

In this study, results suggest that lncRNA SNHG12 expression is significantly increased in DRGs from rats with SNI; thereafter, we hypothesised that lncRNA SNHG12 participates in the initiation or progression of SNI-induced neuropathic pain. The results of qRT-PCR indicated that lncRNA SNHG12 expression was upregulated in the dorsal root ganglia (DRGs) of SNI rat models in a time-dependent manner. RNA-FISH (fluorescence in situ hybridization) results showed that lncRNA SNHG12 was primarily located in the cytoplasm of PC12 cells, suggesting that lncRNA SNHG12 might participate in the cellular progression of PC12 cells. We generated lncRNA SNHG12 knockdown SNI rat models. The results from paw withdrawal threshold (PWT) and paw withdrawal latency (PWL) tests indicated that downregulation of lncRNA SNHG12 attenuated neuropathic pain. Furthermore, lncRNA SNHG12 knockdown decreased the expression of interleukin (IL)-1β, IL-6 and tumour necrosis factor α (TNF-α) in the DRGs in SNI rat models. Through conducting bioinformatics analysis, RNA pull-down, and luciferase reporter gene assays, it was found that lncRNA SNHG12 could regulate RAN23D expression through sponging miR-494-3p. Furthermore, the study results suggested that lncRNA SNHG12 expression in PC12 cells could be transcriptionally regulated by Kruppel Like Factor 2 (KLF2). This study identified a novel KLF2/lncRNA SNHG12/miR-494-3p/RAD23B axis in SNI-induced neuropathic pain. These results might provide new insight for developing diagnosis or therapeutic targets for SNI-induced neuropathic pain in the future.

## Results

### LncRNA SNHG12 expression is upregulated in the DRGs of SNI rats

**Fig. 1:**
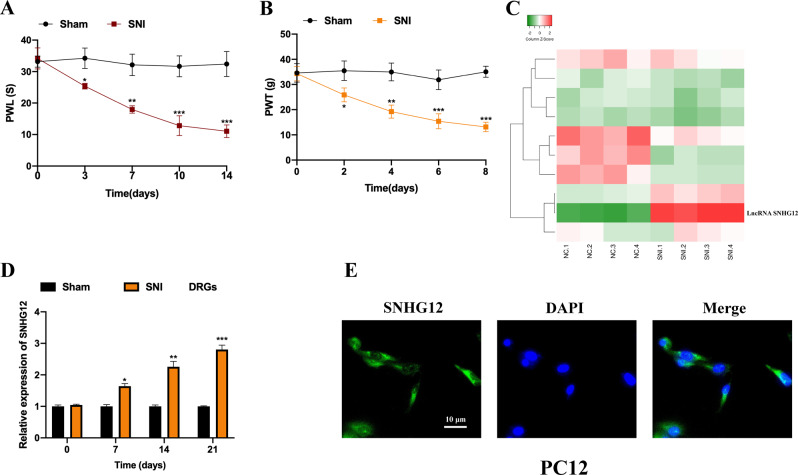
LncRNA SNHG12 expression is upregulated in the DRGs of SNI rats. **A** PWL and **B** PWT assays were conducted to examine the SNI and Sham rats. **C** Microassay analysis results of lncRNA expression profile of the DRGs tissues from SNI or Sham group rats. **D** LncRNA SNHG12 expression in DRGs from SNI or Sham group rats was detected by qRT-PCR. **E** Cell distribution of SNHG12 in PC12 cells was determined by an RNA-FISH assay. Experimental data are presented as mean ± SD. *P < 0.05, **P < 0.01, ***P < 0.001.

To investigate the functional role of lncRNAs in neuropathic pain progression, we generated neuropathic pain rat models using the SNI method. First, PWL and PWT assays were performed to assess model construction. It was observed that both PWL (Fig. 1A) and PWT (Fig. 1B) were markedly decreased in the SNI group, but no significant changes were observed in the sham group. Subsequently, we collected four pairs of DRGs from the SNI group and Sham group for microassay analysis. As shown in Fig. 1C, it was observed that the change in lncRNA SNHG12 expression was significant. Furthermore, results of qRT-PCR showed that lncRNA SNHG12 expression in the DRG tissues from the SNI group increased in a time-dependent manner, when compared with those from the Sham group (Fig. 1D). The distribution of lncRNA SNHG12 in PC12 cells was determined using an RNA-FISH assay, and we observed that lncRNA SNHG12 was mainly located in the cytoplasm (Fig. 1E), suggesting that lncRNA SNHG12 might possess a functional role. These results indicate that lncRNA SNHG12 might participate in neuropathic pain progression originating from DRG tissue.

### LncRNA SNHG12 knockdown represses SNI-induced neuropathic pain

**Fig. 2:**
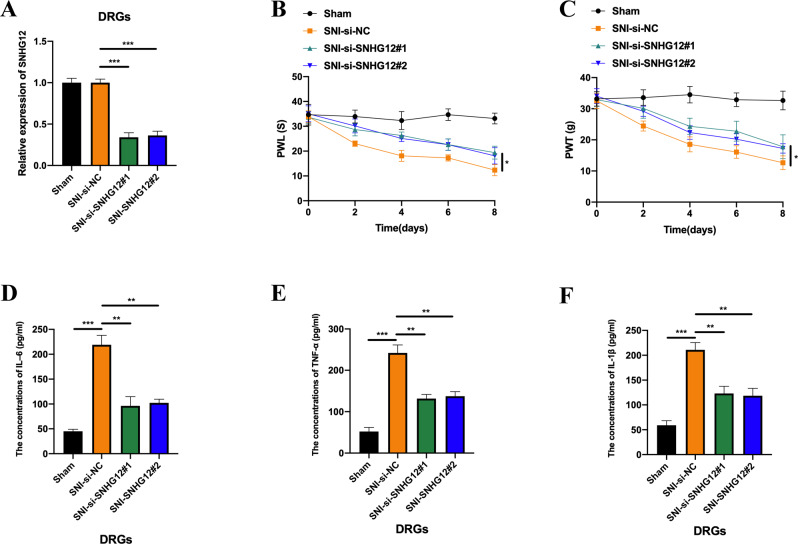
LncRNA SNHG12 knockdown represses SNI induced neuropathic pain. **A** The relative expression level of lncRNA SNHG12 in DRGs from SNI rats pre-intrathecally injected with si-NC, si-SNHG12#1, and si-SNHG12#2. **B** PWL or (**C**) PWT assays were performed to assess the neuropathic pain of rats from the sham or lncRNA SNHG12 knockdown SNI groups. The levels of IL–6 (**D**), TNF–α (**E**), and IL-1β (**F**) in the DRGs of rats from the sham or lncRNA SNHG12 knockdown SNI groups were assessed by ELISA. Experimental data are presented as mean ± SD. *P < 0.05, **P < 0.01, ***P < 0.001.

Here, we generated lncRNA SNHG12 downregulated rat models by injecting si-NC, si-SNHG12#1, and si-SNHG12#2 into SNI rats, to verify the function of lncRNA SNHG12 in SNI-induced neuropathic pain., The intrathecal injection was performed one week after the SNI surgery, and the expression level of lncRNA SNHG12 was detected a week after injection. LncRNA SNHG12 expression was markedly decreased in the DRGs of rats from the SNI group compared with the Sham group after small interfering RNA (siRNA) administration (Fig. 2A). Next, the neuropathic pain behaviours were assessed by PWL and PWT assays. It was observed that lncRNA SNHG12 knockdown significantly attenuated SNI-induced neuropathic pain (Fig. 2B, C). Furthermore, we performed an ELISA assay to quantify neuropathic pain-associated inflammatory cytokines (IL-6, TNF-α, and IL-1β) in the DRGs. LncRNA knockdown markedly inhibited the concentrations of IL-6, TNF-α, and IL-1β in the DRGs of SNI rats (Fig. 2D, E). Based on this evidence, we concluded that lncRNA SNHG12 knockdown attenuated SNI-induced neuropathic pain in rats.

### KLF2 mediates lncRNA SNHG12 expression in PC12 cells

**Fig. 3:**
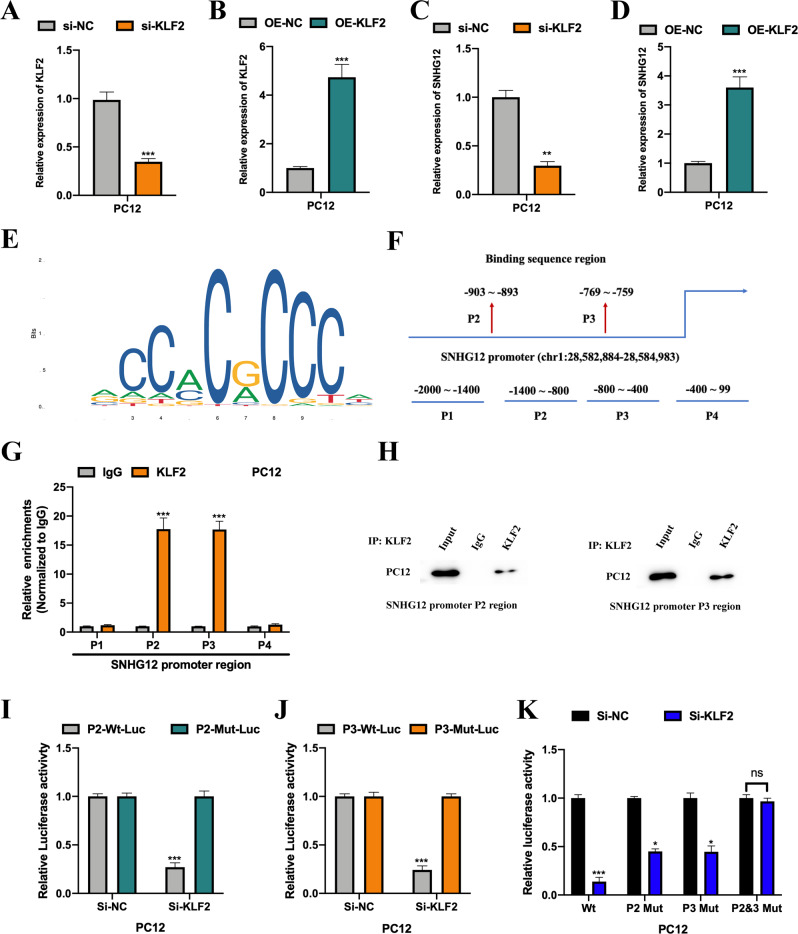
KLF2 mediates lncRNA SNHG12 expression in PC12 cells. The relative expression level of KLF2 in KLF2 downregulated (**A**) or upregulated (**B**) PC12 cells was measured by qRT-PCR. The relative expression levels of lncRNA SNHG12 in KLF2 downregulated (**C**) or upregulated (**D**) PC12 cells were measured by qRT-PCR. **E** The predicted binding sequence of KLF2 is shown. **F** The putative binding regions of the SNHG12 promoter are presented. ChIP assay followed with qRT-PCR (**G**) and western blot (**H**) were conducted to evaluate the enrichment of SNHG12 promoter regions (P1–P4) in anti-IgG or anti-KLF2 bounds. **I**–**K** Luciferase reporter gene assay was performed to verify the interactions between KLF2 and the P2 or P3 promoter regions of SNHG12 in PC12 cells. Experimental data are presented as mean ± SD. *P < 0.05, **P < 0.01, ***P < 0.001.

Accumulating evidence suggests that transcriptional factors play an essential role in the regulation of lncRNAs [22–24]. Using the JASPAR database of interest, we noticed that KLF2 might transcriptionally regulate lncRNA SNHG12 expression. Therefore, we constructed cell models with down- and up-regulated KLF2. The transfection efficiencies were detected (Fig. 3A, B), and we observed that KLF2 could positively regulate lncRNA SNHG12 expression in PC12 cells (Fig. 3C, D), indicating that KLF2 might transcriptionally regulate lncRNA SNHG12. The predicted binding sequences between KLF2 (Fig. 3E) and the promoter regions of SNHG12 (Fig. 3F) were presented. Results of the ChIP assay showed that P2 and P3 regions of SNHG12 promoter could be significantly enriched by anti-KLF2 compared with anti-IgG (Fig. 3G, H). Subsequently, results of the luciferase reporter assay verified the interaction between KLF2 and the P2 and P3 regions of the SNHG12 promoter in PC12 cells (Fig. 3I–K). This suggested that KLF2 could transcriptionally regulate lncRNA SNHG12 expression.

### MiR-494-3p is a molecular sponge for lncRNA SNHG12

**Fig. 4:**
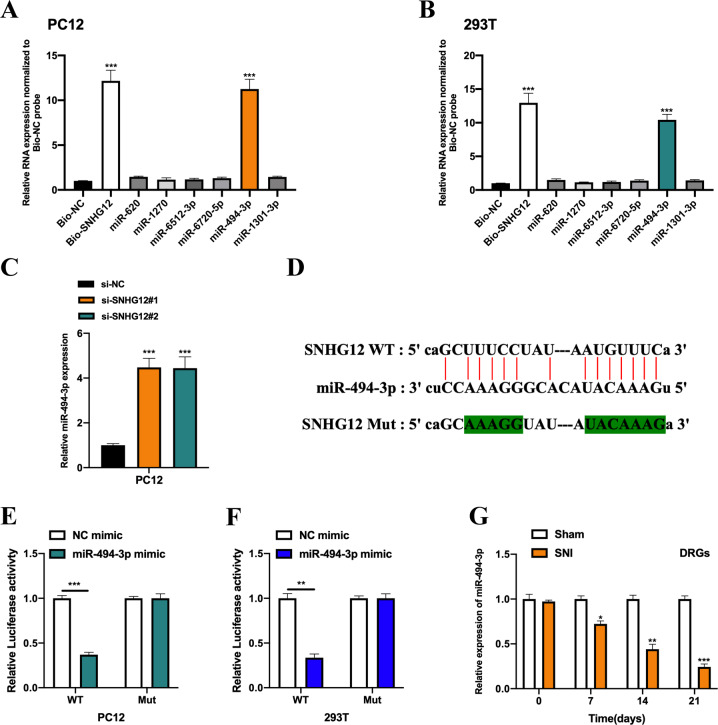
MiR-494-3p is a molecular sponge for lncRNA SNHG12. Biotinylated RNA pull-down assay followed by qRT-PCR assay was performed to assess relative expression levels of putative miRNA targets of lncRNA SNHG12 in PC12 cells (**A**) and 293 T cells (**B**). **C** The relative expression level of miR-494-3p in lncRNA SNHG12 downregulated PC12 cells was measured by qRT-PCR. **D** Predicted binding sequences between lncRNA SNHG12 and miR-494-3p are shown. The binding possibility between lncRNA SNHG12 and miR-494-3p was verified by a luciferase reporter gene assay in PC12 cell (**E**) and 293 T cell (**F**). **G** The relative expression level of miR-494-3p in the DRGs of rats from the Sham or SNI groups was measured by qRT-PCR. Experimental data are presented as mean ± SD. *P < 0.05, **P < 0.01, ***P < 0.001.

To uncover the downstream molecular mechanisms of lncRNA SNHG12 underlying SNI-induced neuropathic pain development, we conducted bioinformatics analysis using the ENCORI database, and six miRNAs were predicted. A biotinylated RNA pull-down assay was performed to verify the putative targets, and it was observed that the bio-SNHG12 probe significantly enriched miR-494-3p both in PC12 and 293 T cells (Figs. 4A and 3B). In addition, it was observed that the expression levels of miR-494-3p in lncRNA SNHG12 downregulated PC12 cells were increased (Fig. 4C). The putative binding sequences between lncRNA SNHG12 and miR-494-3p were synthesised (Fig. 4D), and the interactions between lncRNA SNHG12 and miR-494-3p were subsequently verified by a luciferase reporter gene assay in PC12 cells (Fig. 4E) and 293 T cells (Fig. 4F) as indicated. Furthermore, miR-494-3p expression in SNI rats decreased in a time-dependent manner compared with rats from the Sham group (Fig. 4G).

### MiR-494-3p attenuates SNI-induced neuropathic pain

**Fig. 5:**
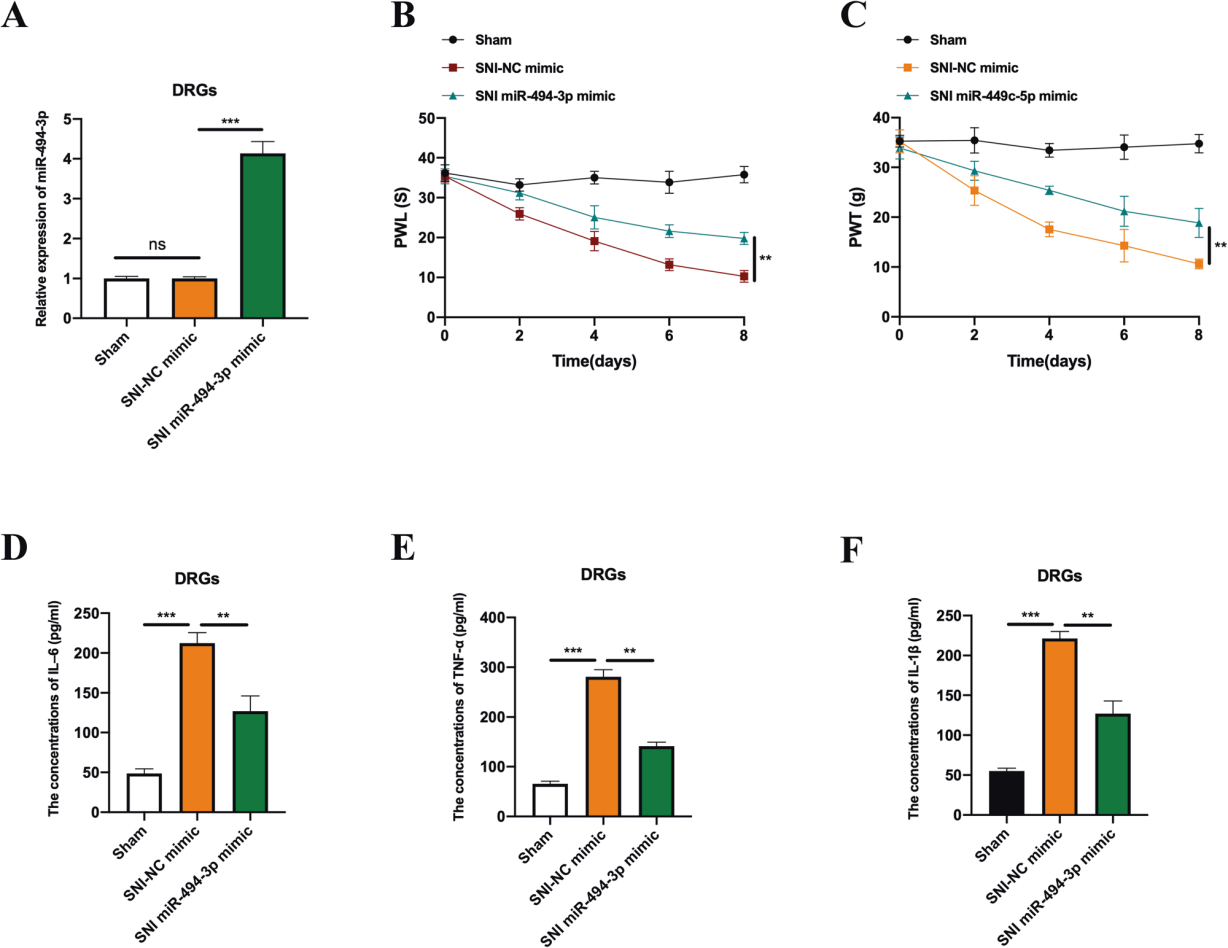
MiR-494-3p attenuates SNI-induced neuropathic pain. **A** The relative expression level of miR-494-3p, in the DRGs from SNI rats pre-intrathecally injected with NC mimic and miR-494-3p mimic was measured by qRT-PCR. **B** PWL and (**C**) PWT assays were performed to assess the neuropathic pain of rats from the sham and miR-494-3p overexpressed SNI groups. The levels of IL–6 (**D**), TNF–α (**E**), and IL-1β (**F**) in the DRGs of rats from the sham and miR-494-3p overexpressed SNI groups were assessed by ELISA assay. Experimental data are presented as mean ± SD. **P < 0.01, ***P < 0.001.

To verify the functional roles of miR-494-3p, we intrathecally introduced NC and miR-494-3p mimics into SNI rats. Relative expression levels of miR-494-3p in the DRGs from SNI rats and Sham rats were measured by qRT-PCR (Fig. 5A), and PWL and PWT assays performed. We observed that miR-494-3p could attenuate SNI-induced neuropathic pain (Fig. 5B, C). Furthermore, overexpression of miR-494-3p could repress the concentrations of IL-6, TNF-α, and IL-1β in the DRGs of SNI rats (Fig. 5D–F).

### MiR-494-3p directly targets RAD23B

**Fig. 6:**
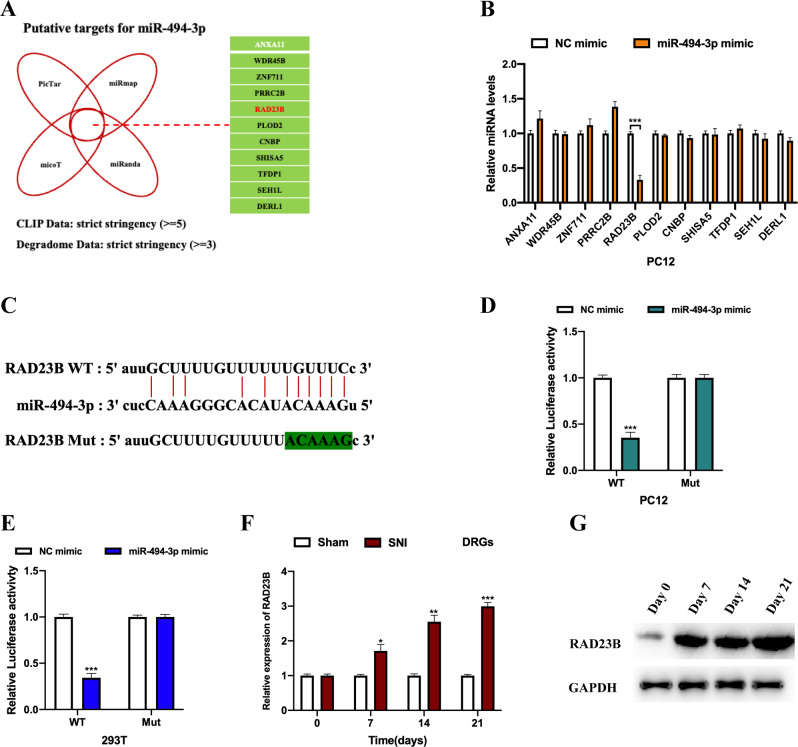
MiR-494-3p directly targets RAD23B. **A** The intersection results of the bioinformatics analysis of miR-494-3p potential targets are illustrated in a scheme. **B** Relative expression levels of putative mRNA targets in miR-494-3p overexpressed PC12 cells were measured by qRT-PCR. **C** Predicted binding sequences between RAD23B and miR-494-3p are shown. The binding possibility between RAD23B and miR-494-3p was verified by a luciferase reporter gene assay in microglial cells (**D**) and 293 T cells (**E**). The relative expression of RAD23B in the DRGs isolated from SNI or Sham rats at the indicated time points was assessed by qRT-PCR (**F**) and western blot (**G**). Experimental data are presented as mean ± SD. *P < 0.05, **P < 0.01, ***P < 0.001.

Subsequently, we investigated the downstream targets of miR-494-3p using bioinformatics analysis. A schematic illustration is shown in Fig. 6A. The results of qRT-PCR demonstrated that miR-494-3p could markedly downregulate RAD23 homologue B, nucleotide excision repair protein (RAD23B) expression in PC12 cells (Fig. 6B), suggesting that RAD23B might be the mRNA target for miR-494-3p. The putative binding sequences between RAD23B and miR-494-3p were synthesised and presented (Fig. 6C). The interactions between RAD23B and miR-494-3p were verified by a luciferase reporter gene assay in PC12 (Fig. 6D) and 293 T cells (Fig. 6E). Moreover, it was observed that RAD23B expression in the DRGs of SNI rats increased in a time-dependent manner compared with rats from the Sham group (Fig. 6F, G).

### LncRNA SNHG12 play its functional role in SNI-induced neuropathic pain through miR-494-3p/RAD23B axis

**Fig. 7:**
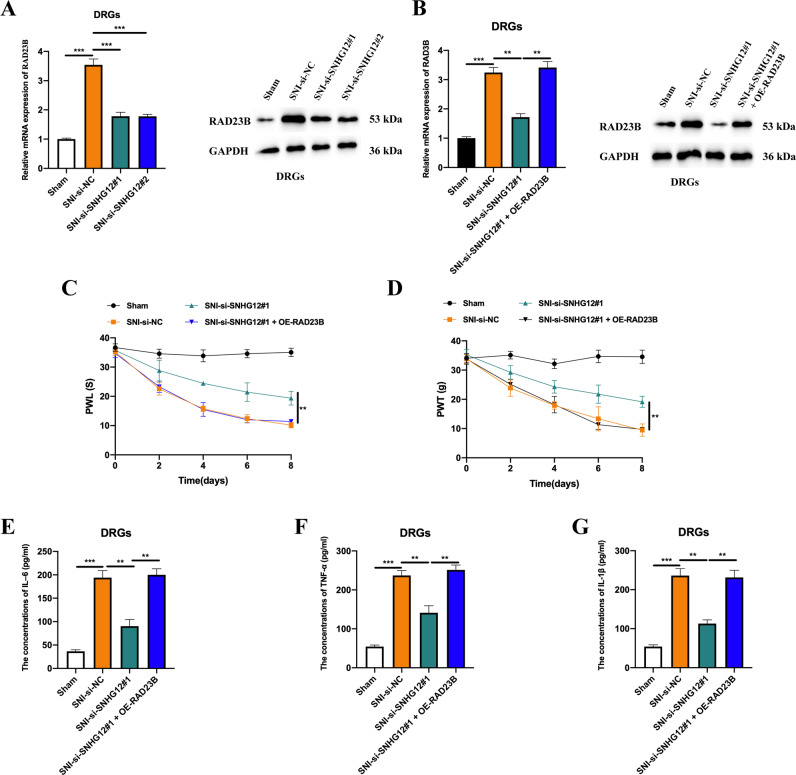
LncRNA SNHG12 plays a functional role in SNI-induced neuropathic pain through the miR-494-3p/RAD23B axis. **A** The mRNA and protein levels of RAD23B in the DRGs from SNI rats pre-intrathecally injected with si-NC, si-SNHG12#1, and si-SNHG12#2. **B** The mRNA and protein expression levels of RAD23B in the DRGs from SNI rats pre-intrathecally injected with si-NC, si-SNHG12#1, and si-SNHG12#1 + OE-RAD23B. **C** PWL or (**D**) PWT assays were performed to assess the neuropathic pain of rats from the sham and pre-treated SNI groups. The levels of IL–6 (**E**), TNF–α (**F**), and IL-1β (**G**) in the DRGs of rats from the sham and the pre-treated SNI groups were assessed by ELISA. Experimental data are presented as mean ± SD. **P < 0.01, ***P < 0.001.

To elucidate whether lncRNA SNHG12 mediates SNI-induced neuropathic pain through regulation of RAD23B, we measured the expression level of RAD23B in SNI rats that had been administrated lncRNA SNHG12 siRNAs, and found it was decreased compared with normal SNI rats (Fig. 7A). Subsequently, we intrathecally injected a RAD23B overexpression vector (OE-RAD23B) into lncRNA SNHG12 knockdown SNI rats, and determined transfection efficiency by qRT-PCR and western blot assays (Fig. 7B). RAD23B overexpression could attenuate the inhibitive effect of lncRNA SNHG12 knockdown on SNI-induced neuropathic pain (Fig. 7C, D), and rescue the repressive phenomenon of lncRNA SNHG12 knockdown on the concentrations of IL-6, TNF-α, and IL-1β in the spinal cord of SNI rats (Fig. 7E–G). Based on these results, we concluded that RAD23B is responsible for the functional role of lncRNA SNHG12 in SNI-induced neuropathic pain.

## Discussion

The different distributions of lncRNA in the nervous system occurs to different results. The dysfunctions of lncRNAs in DRG primary sensory neurons, the dorsal horn of the spinal cord, and peripheral nerves could cause neuropathic pain in different pain-related regions [25]. In this study, we investigated the role of lncRNA in SNI rat models. Based on the results obtained and the essential role of lncRNA SNHG12 in multiple cellular progression, we hypothesised that lncRNA SNHG12 might be involved in the initiation or progression of SNI-induced neuropathic pain.

Firstly, this study successfully constructed SCI rat models, where lncRNA SNHG12 expression was upregulated in the DRGs of rat models, and was primarily distributed in the cytoplasm of PC12 cells. Subsequently, the study results indicated that lncRNA SNHG12 knockdown attenuated the neuropathic pain induced by SNI. The SNI-induced neuropathic pain could be categorised into two sections: (1) the neuropathic pain induced by the primary injury, which referred to the traumatic mechanical damage that causes cell death, and (2) the neuropathic pain induced by the secondary injury, which referred to that due to inflammation [26–28]. Thereafter, we investigated the impact of lncRNA SNHG12 knockdown on the expression level of inflammation factors (IL-6, TNF-α, and IL-1β) in DRGs. ELISA results showed that lncRNA SNHG12 knockdown decreased the expression levels of IL-6, TNF-α, and IL-1β in the DRGs of SNI rat models.

Secondly, we investigated the upstream regulator of lncRNA SNHG12. Accumulating evidence suggests that transcription regulators play a crucial role in modulating the mechanical or biological role of lncRNA [29–31]. This present research indicated that lncRNA SNHG12 expression in PC12 cells could be regulated by KLF2. Thus, we investigated the downstream mechanisms of lncRNA SNHG12 using PC12 cells. The results of bioinformatics analysis, RNA pull-down, ChIP, and luciferase reporter gene assays suggested that lncRNA SNHG12 regulated RAD23B expression via targeting miR-494-3p. We elucidated the functional role of miR-494-3p in SNI-induced neuropathic pain, and we found that lncRNA SNHG12 played its role in SNI-induced neuropathic pain through regulating RAD23B protein expression in the DRGs of SNI rat models. This mechanistically renewed the profile of lncRNA SNHG12.

In summary, this study demonstrated that, while lncRNA SNHG12 expression was upregulated in the DRGs of rat models, lncRNA SNHG12 knockdown attenuated SNI-induced neuropathic pain, and decreased the expression levels of IL-6, TNF-α, and IL-1β in the DRGs of SNI rat models. We also identified the up- and down-stream factors involved in lncRNA SNHG12-mediated progression of SNI-induced neuropathic pain. A novel KLF2/lncRNA SNHG12/miR-494-3p/RAD23B axis might provide new insight for developing novel diagnosis or therapeutic targets for neuropathic pain.

## Materials and methods

### Cell culture and transfection

The cells (PC12 cells and 293 T cells) used in the current study were commercially obtained from the American Type Culture Collection (Manassas, USA). We used DMEM (Lonza, USA) supplied with 10% FBS (Invitrogen, USA) to culture cells in a humid environment with 5% CO_2_ at 37 °C. Transfections were performed using Lipofectamine 2000 reagent (Invitrogen, USA), according to the manufacturer’s instructions.

### Real-time quantitative fluorescence PCR (qRT-PCR)

The cells and DRGs from rats were lysed using Trizol reagent (Thermofisher, USA). A SuperScript IV reverse transcriptase (Thermofisher, USA) was used to reversely transcribe RNAs into cDNAs. The expression level of lncRNA SNHG12 was measured using a SYBR Green kit (Solarbio, China), and GAPDH was used as an internal control. For the mRNA detection, a TaqMan MicroRNA assay kit (Thermofisher, USA) was used, and U6 served as an internal control. Primers used in this study are as follows: lncRNA SNHG12: Forward 5ʹ-GGT GCT CCA GGC AAT AACT-3ʹ, Reverse 5ʹ-CTC CCA TAC AGT CCG AAC AT-3ʹ; miR-494-3p: Forward 5ʹ- GAA ACA TAC ACG GGA AAC C -3ʹ, Reverse 5ʹ- GTG CAG GGT CCG AGG T -3ʹ; RAD23B: Forward 5ʹ- CTT CCT CCA CCA CCA CAA CT -3ʹ, Reverse 5ʹ- GGT GTC TCT GCT GGC TTT TC -3ʹ; KLF2: F 5ʹ- TTC GGT CTC TTC GAC GAC G-3ʹ, R 5ʹ- TGC GAA CTC TTG GTG TAG GTC-3ʹ; U6: F 5ʹ-CTCGCTTCGGCAGCACATA-3ʹ, R 5ʹ-CGAATTTGCGTGTCATCCT-3ʹ; GAPDH: Forward 5ʹ-ACA ACA GCC TCA AGA TCA TCA G-3ʹ, Reverse 5ʹ-GGT CCA CCA CTG ACA CGT TG-3ʹ.

### Microarray analysis

The microarray study analysed the differential lncRNAs expression profiles in DRGs tissues from SNI rat models and normal control rats. Genes were considered differentially expressed if *P* < 0.05 and there was more than a two-fold change. The “Expression” function in the ‘Heatmapper’ package (http://www.heatmapper.ca/expression/) was used to create the heatmap of lncRNAs expressions.

### RNA-fluorescence in situ hybridisation (FISH)

For the cellular distribution of SNHG12 in PC12 cells, an RNA-FISH assay was performed. The SNHG12 probe was commercially obtained from AXL-bio (Guangzhou, China). We cultured the cells in a 6-well plate and added 4% paraformaldehyde for 30 min. The cells were fixed with 0.5% Triton X-100 (20 min). Subsequently, probes were added to the cells, followed by anti-digoxigenin-fluorescein-Fab fragments (Roche Diagnostics, USA). A ProLong Gold Antifade Reagent was used to mount the slide, and the nuclei were stained with DAPI (Invitrogen, USA). The results were visualised using an Olympus confocal microscope (Olympus, Germany).

### Western blot

According to the manufacturer’s instructions, all proteins were collected using a RIPA buffer (Sigma, USA) and separated using SDS-PAGE (Bio-Rad, USA). The proteins were then transferred onto a PVDF membrane (Millipore, USA), and 5% fat-free milk was used to block the membrane. Incubation with the primary antibody happened overnight, followed by incubation with the respective secondary antibody (two hours). The results were visualised using an enhanced ECL system (Tanon, China). The antibodies used, with respective dilutions, were RAD23B (Abcam, ab194273, 1:1000), KLF2 (Abcam, ab203591, 1:1000), IgG (Abcam, ab150165, 1:500), and GAPDH (Abcam, ab8245, 1:5000).

### Chromatin-immunoprecipitation (ChIP)

The ChIP assay was performed using a Magna ChIP Kit (Millipore, USA). After the crosslinking process, the DNA complexes were sonicated into ~200–500 bp fragments, and the fragments immunoprecipitated with primary antibodies. The results were analysed by a qRT-PCR assay. The negative control used was anti-IgG.

### RNA pull-down assay

The biotinylated probes were constructed using an RNA Labelling Mix (Roche). After the bio-probes were introduced into the cells, the magnetic beads were added. Subsequently, the RNA-protein interactions were separated by SDS-PAGE (Bio-Rad Laboratories), and the results analysed by western blot and qRT-PCR assays.

### Luciferase reporter gene assay

For the downstream targets detection, a pmirGLO vector was used to harbour the wild-type (WT) or mutant-type (Mut) sequences of the target gene. Subsequently, vectors were co-transfected with miRNA mimics into the cells, using a Lipofectamine 2000 reagent. For the upstream regulator investigation, the WT or Mut SNHG12 promoter regions (P2/P3) were co-transfected into the cells with pcDNA3.1/RAD23B or pcDNA3.1. The results were analysed with a Dual-Luciferase Report Assay (Promega).

### ELISA

A commercial ELISA kit (Cw Biotech, Beijing, China) was used to detect the levels of IL-1β, IL-6, and TNF-α in the spinal cord tissue isolated from rats, according to the manufacturer’s instructions.

### Behaviour tests

The pain assessments (mechanical allodynia and thermal hyperalgesia) were evaluated by PWT and PWL tests. For the mechanical allodynia assessment, we conducted PWT using the Electric Von Frey device (IITC, USA). A metal mesh floor was placed into a transparent box that housed the rats. The pressure on the plantar surface of the rat’s hindpaw was established using the Electric Von Frey device. For the thermal hyperalgesia assessment, PWL was conducted using the Plantar Test Instrument. Briefly, we recorded and analysed the stimuli and paw withdrawal time, with a cut-off time of 30 s.

### Spared nerve injury (SNI) model

Male Sprague Dawley (SD) rats (5–7 weeks of age) used in the current study were obtained from the Experimental Animal Centre of Southern Medical University (China). We housed the rats in a sterilised cage and gave them free access to food and water. Rats were randomly divided into the SNL and Sham groups (12 rats per group). The L5 spinal nerve was exposed, and this was followed by ligation with 4-0 silk thread for the rats in the SNL group, while the rats in the Sham group were not ligated. We collected spinal cord and DRGs tissues from rat models in accordance with published studies [32, 33]. All procedures conducted on the rats were approved by the Laboratory Animal Ethics Committee of Zhongshan People’s Hospital, according to the International Association for the Study of Pain. For the intrathecal injection, all siRNAs, overexpression lentiviruses, and mimics were transfected into the DRG of rats in the form of an ADV3 adenovirus plasmid with a CMV promoter (Genepharma, China) between EcoRI and BamHI (l × 10^11^ pfu/ml, 20 μl/day).

### Bioinformatics analysis

We predicted the putative miRNA targets of SNHG12 using the ENCORI database (http://starbase.sysu.edu.cn/) (CLIP-seq data: strict stringency (≥5); Class: 8 mer). The mRNA targets of miR-494-3p were analysed using the microT (http://diana.imis.athena-innovation.gr/), miRmap (https://mirmap.ezlab.org/), miRanda (http://www.microrna.org/), and PicTar (https://pictar.mdc-berlin.de/) databases [CLIP-seq data: strict stringency (≥3); degradome data: strict stringency (≥3)]. The prediction of the transcription regulator of SNHG12 was performed using the NCBI (https://www.ncbi.nlm.nih.gov/), UCSC (http://genome.ucsc.edu/), and JASPAR (http://jaspar.genereg.net/) databases.

### Statistical analysis

The statistical analysis of experimental results was performed using the SPSS (SPSS Inc, USA) and GraphPad Prism 7 software (GraphPad, CA). The experiments were conducted in triplicate, and data were presented as mean ± standard deviation (SD). In addition, significant differences between two groups were analysed using the Student’s *t* test, and among three or more groups assessed using One-way ANOVA. A *P* value of <0.05 was considered a significant difference.

## Supplementary information

Unedited WB images

## Data Availability

The datasets used and/or analysed during the current study are available from the corresponding author on reasonable request.
