# Supplementary figures and images for: Identifying a novel KLF2/lncRNA SNHG12/miR-494-3p/RAD23B axis in Spare Nerve Injury-induced neuropathic pain
Source: Cell Death Discov. 2022 May 27;8:272. doi: 10.1038/s41420-022-01060-y (PMC9142504; doi:10.1038/s41420-022-01060-y)

Figure 3-H

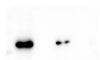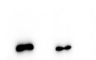

Figure 6-G

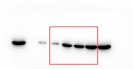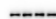

Figure 7-A

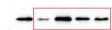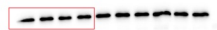

Figure 7-B

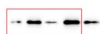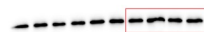

Supplement: Supplementary file 1 — Unedited WB images [file 41420_2022_1060_MOESM1_ESM.pdf]
